# A Pathway-Based Genomic Approach to Identify Medications: Application to Alcohol Use Disorder

**DOI:** 10.3390/brainsci9120381

**Published:** 2019-12-16

**Authors:** Laura B. Ferguson, Shruti Patil, Bailey A. Moskowitz, Igor Ponomarev, Robert A. Harris, Roy D. Mayfield, Robert O. Messing

**Affiliations:** 1 Waggoner Center for Alcohol and Addiction Research, The University of Texas at Austin, Austin, TX 78712, USA; laurazeavin@gmail.com (L.B.F.); shruti.patil@utexas.edu (S.P.); bam2ss@virginia.edu (B.A.M.); harris@austin.utexas.edu (R.A.H.); dayne.mayfield@austin.utexas.edu (R.D.M.); 2 Department of Neuroscience, The University of Texas at Austin, Austin, TX 78712, USA; 3 Department of Neurology, Dell Medical School, The University of Texas at Austin, Austin, TX 78712, USA; 4 Department of Pharmacology and Neuroscience, Texas Tech University Health Sciences Center, Lubbock, TX 79430, USA; igor.ponomarev@ttuhsc.edu

**Keywords:** systems pharmacology, alcohol dependence, alcohol use disorder, gene expression

## Abstract

Chronic, excessive alcohol use alters brain gene expression patterns, which could be important for initiating, maintaining, or progressing the addicted state. It has been proposed that pharmaceuticals with opposing effects on gene expression could treat alcohol use disorder (AUD). Computational strategies comparing gene expression signatures of disease to those of pharmaceuticals show promise for nominating novel treatments. We reasoned that it may be sufficient for a treatment to target the biological pathway rather than lists of individual genes perturbed by AUD. We analyzed published and unpublished transcriptomic data using gene set enrichment of Kyoto Encyclopedia of Genes and Genomes (KEGG) pathways to identify biological pathways disrupted in AUD brain and by compounds in the Library of Network-based Cellular Signatures (LINCS L1000) and Connectivity Map (CMap) databases. Several pathways were consistently disrupted in AUD brain, including an up-regulation of genes within the Complement and Coagulation Cascade, Focal Adhesion, Systemic Lupus Erythematosus, and MAPK signaling, and a down-regulation of genes within the Oxidative Phosphorylation pathway, strengthening evidence for their importance in AUD. Over 200 compounds targeted genes within those pathways in an opposing manner, more than twenty of which have already been shown to affect alcohol consumption, providing confidence in our approach. We created a user-friendly web-interface that researchers can use to identify drugs that target pathways of interest or nominate mechanism of action for drugs. This study demonstrates a unique systems pharmacology approach that can nominate pharmaceuticals that target pathways disrupted in disease states such as AUD and identify compounds that could be repurposed for AUD if sufficient evidence is attained in preclinical studies.

## 1. Introduction

Alcohol Use Disorder (AUD) is a syndrome condition characterized by a number of symptoms and behaviors according to the Diagnostic and Statistical Manual of Mental Disorders (DSM) 5 [1], including a loss of control over alcohol consumption, risky drinking patterns, and a craving for alcohol in its absence. AUD is chronic, relapsing, and highly prevalent, which results in high societal cost and burden [2]. There are currently three FDA-approved treatments for AUD: disulfiram, acamprosate, and naltrexone, none of which are effective for all patients. Therefore, there is a clear, unmet need for new treatment options based on increased understanding of the molecular basis of AUD.

There are numerous studies that have compared gene expression in patients with alcohol dependence with healthy controls across several brain areas implicated in addiction, such as the prefrontal cortex (PFC), basolateral amygdala (BLA), central nucleus of the amygdala (CNA), nucleus accumbens (NAC), hippocampus (HPC), and the ventral tegmental area (VTA) [3,4,5,6,7,8,9,10,11,12,13,14]. These studies point to hundreds of genes and gene co-expression networks that are disrupted in AUD brain and have increased our understanding of the molecular basis of AUD. The findings of these studies have been reviewed [15]. A note on terminology used throughout this manuscript: the gene expression studies used the DSMIV criteria which described two distinct disorders, alcohol abuse and alcohol dependence, whereas the DSM5 integrates the two DSMIV disorders, alcohol abuse and alcohol dependence, into a single disorder called alcohol use disorder (AUD) with mild, moderate, and severe sub-classifications. Therefore, we will use alcohol dependence when discussing the gene expression studies and AUD when discussing the results in a broader context.

There is great potential for genome-wide gene expression data to improve the diagnosis and treatment of complex diseases, and this has been enhanced with the development of the Broad Institute’s Connectivity Map (CMap) and the Library of Network-based Cellular Signatures (LINCS) databases [16,17]. These databases include the gene expression responses to thousands of pharmacological agents applied to human cell lines. Researchers have used these databases to perform in silico gene mapping to identify drugs that are predicted to reverse disease-related gene expression levels and treat various diseases, including several cancers, inflammatory diseases, and brain diseases, among others [18,19,20,21]. The goal of gene mapping is to assess the similarity of the pharmacological-induced gene expression signatures to the gene expression signatures from a biological state of interest, for example, a disease state. Perturbations resulting in highly similar, or highly dissimilar, expression signatures are termed “connected”. We recently used the L1000 database to identify drugs that reduce alcohol intake in HDID-1 mice, a mouse line which was genetically selected for high levels of binge-like drinking [22]. We hypothesized that selection for binge-like drinking behavior alters gene expression patterns in the brain, and that drug perturbations producing anti-correlated patterns may reduce alcohol drinking. The top-ranking drug candidates, terreic acid and pergolide, reduced ethanol consumption and blood alcohol levels in HDID-1 mice [22]. 

The current study differs from previous gene mapping approaches in an important way: instead of targeting individual genes, we sought to target pathways. With this approach, the focus is shifted from single genes to groups of genes known to act together in a biological pathway. Here, we hypothesized that individuals with alcohol dependence have modifications in expression levels of genes within important biological pathways, and that drug perturbations producing opposing effects on the same biological pathways may be beneficial in treating AUD. We defined the pathways that are perturbed in alcohol dependence across six discrete brain areas. We also used L1000 and CMap to define the pathways affected by over 3000 repurpose-able compounds, i.e., those that are currently marketed (launched), in clinical trials, or preclinical compounds with good absorption, distribution, metabolism, and excretion (ADME) properties. Additionally, we created a website where these findings are easily accessible (https://networkmeds.shinyapps.io/rshiny_app/). This resource can be used to nominate drugs that target pathways of interest or to nominate pathways predicted to be affected by a drug for studies of potential mechanisms of action. In addition to the alcohol dependence-specific insights this study provides, we hope that this disease-agnostic tool will be useful to the wider scientific community.

## 2. Materials and Methods

### 2.1. Human Gene Expression Datasets

We included 17 gene expression datasets in our analysis that assessed gene expression levels in postmortem brain tissue from patients with alcohol dependence and healthy controls (Table 1). Fifteen of these datasets were published. The two unpublished datasets (Mayfield, unpublished; GEO accession number pending) are CeA and BLA samples from the same individuals for which gene expression data from the PFC have been previously analyzed and published [5]. These data were processed in the same way as the PFC samples. Briefly, Total RNA from postmortem human CeA and BLA was extracted from frozen tissue, excluding any samples with contaminated or degraded quality (RNA integrity numbers less than 5.0). Ribosomal RNA was depleted using RiboMinus Eukaryote kit for RNA-Seq and confirmed using an Agilent Bioanalyzer. Samples were processed using ABI whole transcriptome library preparation kit and sequenced on the ABI SOLiD 4 system using paired-end reads (35 + 50 bp). Collected reads were processed by the Texas Advanced Computing Center and mapped for sequence reads, allowing two mismatches per 25 bp seed length, against the human reference genome (hg19), to select unique alignments with the highest reproducible mapping. Reads were annotated using the University of California Santa Cruz (UCSC) reference hg19. Gene-level read counts were generated using the Partek Genomics Suite software version 6.12. A minimum of five reads/alignment was used to determine values. Read counts were adjusted for sequencing depth (library size). Detection of differential expression was conducted using the DESeq2 package [23]. For the published studies, we used the differential expression results provided in the publication or by the authors [3,4,5,6,7,11,12,13]. There were two microarray studies for which the differential expression results were not provided in full [9,10]. For these we performed a statistical analysis comparing alcohol-dependent and control groups using the Bioconductor package Limma version 3.40.2 to perform a Bayesian two-tailed *t*-test. A false discovery rate (FDR) for each gene was estimated using the method of Benjamini and Hochberg [24]. Differential expression analysis results were in the form of a table containing a list of gene symbols, fold changes (expression level of the gene in alcohol-dependent relative to control brain), *p* values for the moderated t test statistic, and adjusted *p* values based on the FDR. 

### 2.2. Drug Gene Expression Datasets

We downloaded the differential gene expression signatures for over 20,000 compounds (L1000 level 5 data) from Gene Expression Omnibus (Phase I: GSE92742, Phase II: GSE7013), where each signature is the result of a different drug, dose, cell line, and time point for which differential expression was assessed. To increase the reliability of the L1000 gene expression signatures, we further limited our analysis of level 5 L1000 data to only include signatures with distil_cc_q75 ≥ 0.15, where distil_cc_q75 is a measure of the reproducibility of a signature (see https://clue.io/connectopedia/glossary for more information). 

We downloaded the CMap amplitude data (amplitudeMatrix.txt) from ftp://ftp.broadinstitute.org/distribution/cmap/. Amplitude is a measure of the extent of differential expression of a given probe set. Amplitude *a* is defined as follows:

(1) $$\frac{t-c}{\left(t+c\right)/2}$$

where *t* is the scaled and thresholded average difference value for the treatment and *c* is the thresholded average difference value for the control. Thus, *a* = 0 indicates no differential expression, *a* > 0 indicates increased expression upon treatment, and *a* < 0 indicates decreased expression upon treatment. For example, an amplitude of 0.67 represents a two-fold induction (see https://portals.broadinstitute.org/cmap/help_topics_linkified.jsp for more information).

To maximize the application of our findings to human studies we used information from the Drug Repurposing Hub to highlight the repurposeable compounds from L1000 and CMap, i.e., those that are currently marketed, in clinical trials, or preclinical compounds with good absorption, distribution, metabolism, and excretion (ADME) properties [25]. We identified 2661 unique, repurposeable compounds that were assayed at various doses and time points on different cell lines in L1000 (a total of 156,763 signatures). The CMap database contains 6102 signatures for 1309 unique compounds, and 861 of those are repurposeable.

### 2.3. Pathway and Cell Type Enrichment Analysis 

We performed a gene set enrichment analysis using the fgsea (Fast Gene Set Enrichment Analysis) Bioconductor package version 1.8.0 [26]. The pre-ranked gene set enrichment analysis takes two objects as input: an array of gene statistic values S and a list of query gene sets P. The goal of the analysis is to determine which of the gene sets from P has a non-random behavior. To quantify a co-regulation of genes in a gene set P, Subramanian et al. introduced a gene set enrichment score (ES) that uses gene rankings (values of S) [27]. The more positive the value of ES, the more enriched the gene set is in up-regulated genes; conversely, negative ES corresponds to enrichment in the down-regulated genes. In our analysis, genes from Kyoto Encyclopedia of Genes and Genomes (KEGG) pathways [28] were downloaded from the Broad Institute’s Molecular Signature Database (MSigDB; version 6.2) and were treated as gene sets P. KEGG is an integrated database resource for understanding high-level functions and utilities of the biological system from molecular-level information. KEGG consists of 18 databases, including KEGG pathway in which experimental knowledge on molecular interaction, reaction and relation networks representing systemic functions of the cell and the organism are captured from literature and organized into pathway maps. We used the membership of KEGG pathways in the gene set enrichment analyses conducted in the present study which were included in the MSigDB download. An ES value was calculated for each of the input pathways. As the null distribution for the ES statistic is not known, an ES value was calculated for a number of random gene sets of the same size. Then, a *p* value was estimated as the number of random gene sets with the same or more extreme ES value divided by the total number of generated gene sets [26,27]. The number of permutations was 10,000 for the postmortem gene expression datasets and the CMap profiles, and 1000 for the L1000 profiles due to the high computational demand of the larger database. A false discovery rate (FDR) for each pathway ES score was estimated using the method of Benjamini and Hochberg [21]. The observed ES scores were determined to be significant at FDR ≤ 5%. The ES scores, unadjusted and FDR-adjusted *p* values for each pathway are presented in Table S1 for each dataset.

For the human alcohol dependence gene expression datasets, the t values were treated as the gene statistic values S. Z scores from the L1000 dataset and amplitudes from the CMap dataset were treated as the gene statistic values S. We set minSize = 15, maxSize = 500, nperm = 10,000 in the fgsea function. Because the L1000 database is much larger than CMap, we had to make the following modifications: we performed the analysis on a computer server at UT Austin and ran the R session through the command line. We ran the following R commands before fgsea: setTimeLimit(cpu = Inf, elapsed = Inf, transient = FALSE) and setSessionTimeLimit(cpu = Inf, elapsed = Inf). For the fgsea function we set nperm = 1000 and nproc = 20. For all pathway enrichment analyses, we collapsed similar pathways together using the collapsePathways function. This enabled us to find which biological pathways are perturbed in alcohol-dependent brain tissue and by repurposeable drugs, along with the direction of perturbation (increased or decreased compared with control brain).

We tabulated the significantly perturbed pathways for each human alcohol dataset to identify those most commonly implicated in alcohol dependence. To determine if genes known to be cell type markers were affected disproportionately, we also performed an enrichment analysis for cell type specific datasets using the userlistEnrichment function from the WGCNA package version 1.68 with useBrainLists = TRUE. The cell type marker lists include neurons [29], glutamatergic neurons [30], GABAergic neurons [30], oligodendrocytes [29], microglia [31,32,33,34,35], astrocytes [29,36,37,38], and postsynaptic density [35,39]. Details about the cell type datasets can be found at https://www.rdocumentation.org/packages/WGCNA/versions/1.25-1/topics/userListEnrichment/. The userlistEnrichment function determined the overlap between the cell type marker genes and the differentially expressed genes. We provided the up-regulated differentially expressed genes (positive fold change and nominal *p* < 0.05 for cases versus controls) and the down-regulated differentially expressed genes (negative fold change and nominal *p* < 0.05 for cases versus controls) to determine direction of change for the cell type markers. The userlistEnrichment function uses the hypergeometric distribution to assess the significance of the cell type gene enrichment and Bonferroni correction was applied for multiple testing (Bonferroni-corrected *p* < 0.05 was used for significance). The enrichment *p* values for the cell type markers are presented in Table S2. Enrichment analyses were implemented in R version 3.6.0.

### 2.4. R Shiny Interactive Webpage

To enable accessibility of our results, we built a user-friendly web interface using the shiny package version 1.3.2 [40] in R. Users can select a KEGG pathway or drug of interest from a dropdown list. This information is used to subset the large table of results from the CMap and L1000 fgsea analyses using tidyverse version 1.2.1 [41] and data.table version 1.12.2 [42]. A table subset is output for user viewing based on selected pathway or drug along with a schematic of the relevant pathway (if pathway is selected) and a frequency table which displays the most commonly occurring drug or pathway. We downloaded the pathway images from KEGG using the png package version 0.1–7 [43].

## 3. Results

### 3.1. Pathway and Cell Type Enrichment Analysis of Human Alcohol Gene Expression Datasets

**Figure 1:**
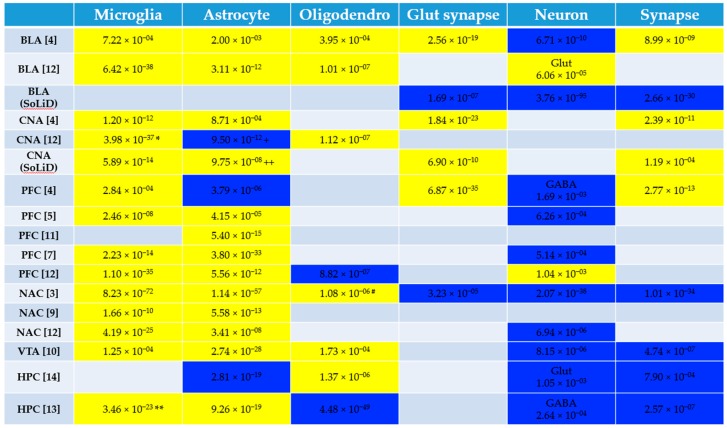
Cell Type Enrichment Results. We determined whether genes preferentially expressed in specific cell types were enriched in the genes differentially expressed between alcohol-dependent and control brain tissue using the userlistEnrichment function from the WGCNA package in R (see Methods). The human alcohol gene expression datasets are the rows (brain region of the dataset is shown in the first column) and the cell types are columns. Yellow indicates that the genes preferentially expressed in the cell type are up-regulated in alcohol-dependent brain tissue and blue indicates genes preferentially expressed in the cell type are down-regulated in alcohol-dependent brain tissue (Bonferroni-corrected *p* < 0.05). The *p* values associated with the enrichment are shown. If a cell type had more than one cell type marker gene list associated with it (from multiple publications, for example), the most significant *p* value is shown in the figure. See Table S2 for the full table of *p* values resulting from the enrichment analysis for all datasets. Some of the cell types were enriched in both the up-regulated and down-regulated datasets. The direction chosen for the figure was based on a more significant enrichment and greater number of enriched datasets for that cell type if applicable. These occurrences are denoted in the figure and described below. * Type I microglial genes were enriched in the down-regulated genes: purple_M4_Microglia(Type1)__CTX (*p* = 4.61 × 10^−5^) and pink_M10_Microglia(Type1)__HumanMeta (*p* = 6.43 × 10^−5^). ** magenta_M8_Microglia(Type2)_MouseMeta genes were enriched in the down-regulated genes (*p* = 3.11 × 10^−7^). + Astrocyte_probably__Cahoy genes were enriched in the up-regulated genes (*p* = 0.000178). ++ brown_M15_Astrocyte__CTX genes were enriched in the down-regulated genes (*p* = 0.00131). **#** Oligodendrocyte_probable__Cahoy genes were enriched in the down-regulated genes (*p* = 9.75 × 10^−5^). Note that Oligodendrocyte_definite__Cahoy genes were enriched in the up-regulated but not down-regulated genes for this dataset. BLA: basolateral amygdala, CNA: central nucleus of the amygdala, PFC: prefrontal cortex, NAC: nucleus accumbens, VTA: ventral tegmental area, HPC: hippocampus, Glut: glutamatergic.

Altogether, there were 17 datasets spanning six brain areas: prefrontal cortex (5), hippocampus (2), nucleus accumbens (3), basolateral amygdala (3), central nucleus of the amygdala (3), and ventral tegmental area (1) (Table 1). The cell type enrichment analysis revealed a consistent up-regulation of microglia-specific genes. Astrocyte-specific genes tended to be up-regulated with three exceptions, while neuronal and synaptic genes were mostly down-regulated, except glutamatergic synaptic genes which were mostly up-regulated in alcohol-dependent brain samples compared with control (Figure 1; Figure S1, Table S2). Oligodendrocyte-specific genes were affected in about half the datasets, sometimes up-regulated and sometimes down-regulated.

We found 58 up-regulated and 36 down-regulated pathways in at least one dataset (Table S1). Out of the 58 up-regulated pathways, 29 occurred in more than one dataset, and four pathways were up-regulated in nearly half of the datasets (8/17) (04610: Complement and coagulation cascades, 04510: Focal adhesion, 04010: MAPK signaling pathway, and 05322: Systemic lupus erythematosus). Out of the 36 down-regulated pathways, 12 occurred in more than one dataset, and one pathway was down-regulated in 6/17 datasets (00190: Oxidative phosphorylation) (Table 2; Table S1). These pathways were disrupted in most brain areas, but no pathway was dysregulated in all brain areas (Table 3), although related pathways might be affected in all brain areas. For example, the focal adhesion pathway was up-regulated in all brain areas except for VTA, but a similar pathway (ECM receptor interaction) was up-regulated in VTA. Some of the most frequently occurring genes in these main pathways include: focal adhesion (*ITGB4*, *VWF*, *AKT1 COL4A1*, *COL6A1*, *COL6A2*, *COL6A3*, *ERBB2*, *LAMB2*, *SHC1*, *ZYX*, *CAV2*, *COL4A2*, *FLNA*, *FN1*, *ITGA5*, *ITGA6*, *KDR*, *VASP*, *ACTN4*, *CAV1*, *FLNB*), complement and coagulation cascade (*C1R*, *CFI*, *SERPINA1*, *SERPING1*, *C1S*, *CFH*, *PLAUR*, *THBD*, *C5AR1*, *C7*, *SERPINA5*), MAPK signaling (*MAPKAPK2*, *AKT1*, *FOS*, *GADD45A*, *GADD45B*, *GNG12*, *HSPB1*, *JUN*, *JUND*, *MKNK2*, *NTRK2*, *ITGA6*, *KDR*, *VASP*, *ACTN4*, *CAV1*, *FLNB*), systemic lupus erythematosus (*C1S*, *C1QC*, *C1R*, *HIST2H2AA3*, *ACTN4*, *C1QB*, *FCGR2A*, *H2AFX*, *H3F3B*, *HIST2H2AC*, *HIST2H2BF*, *HLA-DPA1*, *C4A*, *CD40*, *H2AFJ*, *H3F3A*, *HIST1H2AC*, *HIST1H2AK*, *HIST1H2BE*, *HIST1H2BJ*), oxidative phosphorylation (*ATP6V1E1*, *COX6C*, *CYC1*, *NDUFA1*, *NDUFA4*, *NDUFB5*, *NDUFB6*, *ATP5A1*, *ATP5H*, *ATP5L*, *ATP6V0B*, *ATP6V1A*, *ATP6V1B2*, *COX5A*, *COX6A1*, *COX7A2*, *NDUFA6*, *NDUFA8*, *NDUFAB1*, *NDUFB1*).

### 3.2. Pathway Level Analysis of Drug Gene Expression Datasets

#### 3.2.1. CMap Pathways

**Figure 2:**
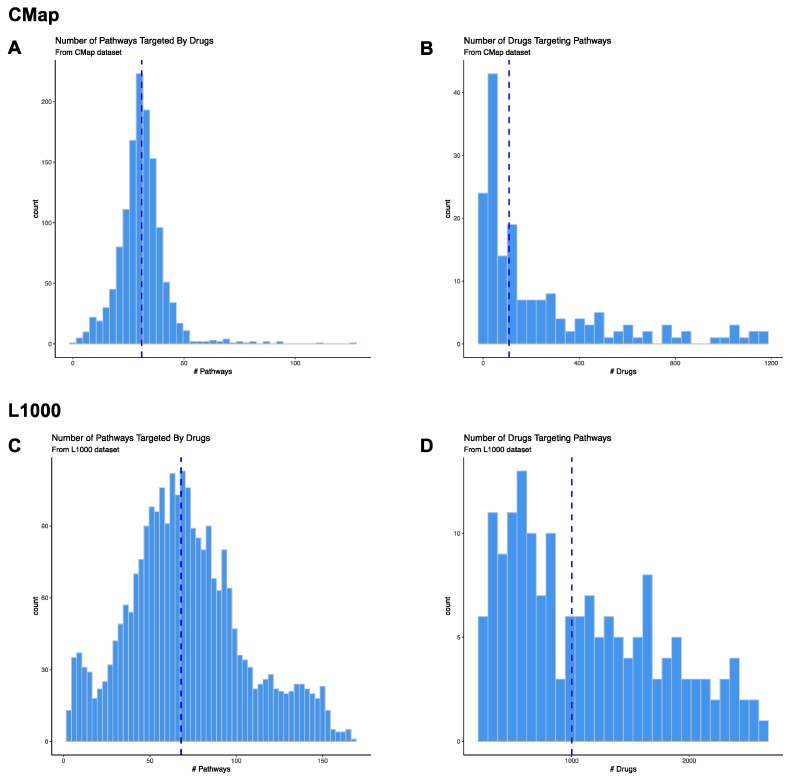
Drug-Pathway Prediction Results. We determined whether genes within KEGG pathways (within the MSigDB v6.2 dataset) were significantly up-regulated or down-regulated by drugs in CMap and L1000 databases using Gene Set Enrichment Analysis (GSEA). We downloaded the drug gene expression signatures for CMap from ftp://ftp.broadinstitute.org/distribution/cmap/ (amplitudeMatrix.txt) and the L1000 signatures from Gene Expression Omnibus (Level 5 data; Phase I: GSE92742, Phase II: GSE7013). Histograms of the number of pathways predicted to be targeted by drugs in CMap (**A**) or L1000 (**C**) databases. Histograms of the number of drugs in CMap (**B**) or L1000 (**D**) databases predicted to target pathways. The blue dashed line represents the median number of pathways in A and C or drugs in B and D.

We assessed 186 KEGG pathways, and 172 (92.5%) of these were predicted to be targeted by drugs in CMap. For the druggable pathways, 1 (0.08%) drug (for Mismatch Repair) to 1169 (89.3%) drugs (for Neuroactive Ligand Receptor Interaction) were predicted to target the pathway (Figure 2A; Table S3).

#### 3.2.2. CMap Drugs

We downloaded gene expression signatures for 1309 unique compounds assayed by CMap. We determined that 861 of these compounds were repurposeable using annotation provided by the Drug Repurposing Hub [25]. We included information on all compounds instead of only restricting our analysis to the repurposeable compounds. Out of the 1309 compounds, 1296 (99%) significantly affected at least one pathway. The compounds were predicted to affect one (0.5%) to 125 (67.2%) unique pathways, with a median of 31 (16.7%) pathways (Figure 2B; Table S3).

#### 3.2.3. L1000 Pathways

We explored 186 KEGG pathways, and 167 (89.8%) of these were predicted to be targeted by drugs in L1000. For the druggable pathways, 214 (8%) drugs (for Sphingolipid Metabolism) to 2599 (97.7%) drugs (for Neuroactive Ligand Receptor Interaction) were predicted to target them (Figure 2C; Table S3).

#### 3.2.4. L1000 Drugs

We downloaded gene expression signatures for 20,629 unique compounds assayed by L1000. We determined that 2661 of these compounds were repurposeable using annotation provided by the Drug Repurposing Hub [25]. Out of the 2661 repurposeable compounds, 2619 (98.4%) significantly affected at least one pathway. The compounds were predicted to affect three (1.5%) to 167 (89.8%) unique pathways, with a median of 68 (36.6%) pathways (Figure 2D; Table S3). Some of the broad-acting compounds that affect most pathways include proteasome inhibitors (bortezomib, MG-132), HDAC inhibitors (vorinostat, trichostatin-A, entinostat), MEK inhibitors (selumetinib, PD-0325901), CDK inhibitors (CGP-60474, PHA-793887) and the control vehicle dimethylsulfoxide (DMSO). Some of the more specific compounds that affect only a few pathways include antibiotics (apramycin, lymecycline, oxacillin, thiamphenicol), acetylcholinesterase inhibitors (rivastigimine, demecarium), sodium channel blocker (benzocaine), adenosine receptor antagonist (etofylline), among others (Table S3).

### 3.3. Candidate Treatment Selection 

Most gene mapping studies that nominate treatments for a disease operate under the reversal hypothesis, which posits that reversing the disease-induced gene expression signature will treat disease phenotypes. More specifically, these approaches look for drugs that up-regulate the genes that are down-regulated in the disease state, and down-regulate genes that are up-regulated in the disease state. We took a similar approach here, but instead of looking for reversal of individual genes, we looked for reversal of disrupted pathways. Following this premise, we searched for compounds that down-regulated the following pathways: 04610: Complement and coagulation cascades, 04510: Focal adhesion, 04010: MAPK signaling pathway, and 05322: Systemic lupus erythematosus, and up-regulated the 00190: Oxidative phosphorylation pathway. 

There were 244 L1000 compounds predicted to target the full combination of pathways in the desired direction across numerous drug classes, including EGFR inhibitors, topoisomerase inhibitors, MEK inhibitors, tyrosine kinase inhibitors, HDAC inhibitor, glucocorticoid receptor agonists, dopamine receptor antagonists, PARP inhibitors, and phosphodiesterase inhibitors (Table S4). Many have already been shown to affect alcohol consumption in patients or animal models, including ondansetron [44], probenecid [45], rolipram [46], clonidine [47], buspirone [48], decitabine [49], GS-39783 [50], rosiglitazone, trichostatin-A [51,52], vorinostat (also called SAHA) [49,53], valproic acid [54], TPCA-1 [55], tamoxifen [56], amlexanox [57], pregnenolone [58], nifedipine [59], metergoline [60], and NVP-TAE684 [61]. We filtered the remaining compounds for those that are currently marketed or in clinical trials and predicted to get into brain. This resulted in 37 candidate compounds. We searched the literature for each of the 37 compounds to gather information on toxicity, contraindications, and effects on neuropsychiatric traits. This resulted in a prioritized list of eight compounds (Table 4).

There were no CMap compounds predicted to target the full combination of pathways in the desired direction. There were 32 compounds predicted to target one of the pathways in the desired direction (Table S5). Of these, only mycophenolic acid was predicted to target more than one of the desired pathways (down-regulate systemic lupus erythematosus and up-regulate oxidative phosphorylation pathway). Eight of the drugs were also predicted to reverse all five pathways in the L1000 analysis: cytochalasin B, fulvestrant, geldanamycin, genistein, MG-132, mycophenolic acid, sirolimus, and trichostatin A. 

### 3.4. Interactive Website to Identify Compounds Predicted to Target Biological Pathways of Interest

The development of R Shiny environments [40] enable R-based user-friendly web interfaces. Using these tools, we offer a novel platform that can be useful on two fronts: (1) identifying relevant drugs and genes of interest based on user-selected biological pathways, (2) generating hypotheses for a drug’s mechanism of action. This app (https://networkmeds.shinyapps.io/rshiny_app/) is disease-agnostic and could apply to any biological state of interest. Therefore, it may appeal to a broad range of researchers including biologists, pharmacologists, chemists and clinical scientists.

## 4. Discussion

We previously used the top differentially expressed genes between HDID and control HS mice to capture molecular changes important for the HDID phenotype of intense binge-like drinking [22]. An alternative approach is to compare drug and disease signatures at the gene-set level (i.e., pathways or gene network/modules), rather than individual genes, which might identify compounds with greater selectivity for reducing ethanol consumption [21,22,62,63]. This approach would also help to mitigate the volatility of drug candidate predictions that might be driven by a handful of genes. Here, we identified biological pathways affected in alcohol-dependent patients across six brain areas. We then mined the CMap and L1000 databases to identify biological pathways affected by drug treatments, and used this information to determine drugs predicted to target many of the pathways disrupted in alcohol dependence.

Our study highlights the pathways and cell types implicated in alcohol dependence across multiple datasets. Half of the up-regulated pathways and one third of the down-regulated pathways occurred in multiple datasets. The cell type results were strikingly consistent across studies. There was a general up-regulation of glial transcripts (microglia ≫ astrocytes). The direction of change for astrocyte- and oligodendrocyte-specific genes were somewhat variable (sometimes up-regulated and sometimes down-regulated) which could indicate that subtypes of these cells are being differentially regulated in alcohol dependence. Notably, many of the up-regulated pathways were immune-related: complement and coagulation cascade, cytokine-cytokine receptor interaction, leukocyte transendothelial migration, natural killer cell mediated cytotoxicity, antigen processing and presentation, the JAK STAT signaling pathway, and the toll-like receptor signaling pathway. The up-regulation of these pathways and microglial genes across brain areas and datasets reinforces the hypothesis that neuroinflammation plays an important role in AUD [64,65,66,67,68,69].

Pathways related to the extracellular matrix (focal adhesion, ECM receptor interaction, and cell adhesion molecules (CAM) pathways) were also prominent in the up-regulated pathways for all brain areas. The importance of the extracellular matrix in learning and memory processes and substance use disorders including AUD has become an active area of research in the last decade [70,71]. Perineuronal nets (PNNs), ECM structures that surround neurons, are increased after binge-like drinking in mouse insula [72], and disrupting PNNs in the insula make mice more sensitive to quinine-adulterated ethanol but not ethanol alone, suggesting that PNNs are important for developing aversion-resistant drinking [73]. Disrupting PNNs in the amygdala combined with extinction training erases drug reward memory (cocaine and morphine, but not food) [74]. Mice missing the extrasynaptic protease MMP-9 display lower motivation for alcohol and impaired alcohol seeking during withdrawal [75]. Genome-wide association studies link polymorphisms in genes that encode ECM components (COL6A3, COL8A1, COL4A3) with AUD and alcohol responsiveness [76,77,78]. Additionally, gene expression patterns in mouse VTA after decitabine treatment (a drug that lowered ethanol intake) highlighted biological categories associated with ECM [49]. Collectively, these results argue that ECM structural components and remodeling enzymes are important determinants of alcohol-induced neuroadaptations.

The majority of tissue samples used in the original studies are independent and were obtained from the New South Wales Tissue Resource Centre at the University of Sydney with the exception of the Zhou 2011 [14] samples which are from the University of Miami Brain Bank. Studies assaying populations other than Australian Caucasian subjects are necessary to determine whether these results extend to those populations as well. One limitation of postmortem datasets is the “chicken and the egg” conundrum, meaning that it is impossible to determine whether differences between alcohol-dependent and control populations predate alcohol use or are from years of excessive alcohol use or management of the disorder. Along those lines, it is difficult to disentangle gene expression changes driving the maladaptive behaviors associated with addiction that should be “reversed” and homeostatic gene expression changes that are beneficial and should be “mimicked”. The KEGG database also includes some “directional” information regarding how the gene products in the pathway affect one another when this is known (including activation/inhibition, phosphorylation/dephosphorylation, methylation, binding, and other types of interactions). Here we highlight the KEGG pathways for which the genes in that pathway occur at the extremes of a list of genes ranked by the test statistic resulting from the comparison of alcohol-dependent and control brain tissue. Importantly, the functional outcome of the pathway cannot be predicted. For example, it would not be a correct interpretation of our results to say that there is a lower level of oxidative phosphorylation in alcohol-dependent brain tissue. The correct interpretation is that many of the genes that are down-regulated in postmortem alcohol-dependent brain tissue are involved in the oxidative phosphorylation pathway. We chose to assess the pathways associated with the up-regulated and down-regulated genes in our analysis. However, the data could be reanalyzed using the absolute change between the alcohol-dependent and control brain tissue to remove information on direction and only consider the magnitude of change. The data could also be reanalyzed in the future using pathway information from other curated databases including BioCarta, Reactome, WikiPathways, and others.

We identified over 240 drugs that target the disrupted pathways in a direction proposed to treat AUD. More than 20 of these compounds are known to alter alcohol drinking, which increases confidence in the ability of this approach to identify useful drug candidates. The candidates we prioritize in Table 4 include five compounds already marketed for indications other than AUD and three in clinical trials for other indications. Methylene blue, a cationic thiazine dye with redox-cycling properties and selectivity for the nervous system, has been used to treat pain, malaria, and neuropsychiatric disorders for more than a century [79,80]. Rucaparib is a first-in-class Poly(ADP-ribose) polymerase (PARP) inhibitor being developed as an anti-cancer agent. Because PARP increases in the nucleus accumbens with cocaine exposure and PARP-1 induction in accumbens mediates enhanced behavioral responses to cocaine [81], it is plausible that inhibiting PARP could treat stimulant and alcohol use disorders. Azelastine and ketotifen are histamine blockers used to treat itchy eyes, asthma, and other allergic disorders. ALX-5407 is a glycine transporter-1 inhibitor. In preclinical models, inhibition of GlyT1 can suppress alcohol and cocaine intake and reinstatement in rats [82,83], but ALX-5407 has not yet been tested. Diflunisal is a nonsteroidal anti-inflammatory drug (NSAID) used to treat rheumatoid arthritis and osteoarthritis, and can decrease in Aβ1-42 levels when given orally to Tg2576 mice [84]. Rilmenidine is an adrenergic receptor agonist used to treat hypertension. There is evidence that rilmenidine might protect serotonin neurons from MDMA-induced toxicity in vitro [85]. It can attenuate the signs of Huntington’s disease and reduce levels of a mutant huntingtin fragment in a mice mouse model [86] and early clinical evidence appears promising for repurposing rilmenidine for Huntington’s disease [87]. Indirubin is a cyclin-dependent kinase (CDK) and glycogen synthase kinase-3 (GSK-3) inhibitor used to treat leukemia in China and is in clinical trials for psoriasis (NCT01735864, NCT02088281, NCT01445886) and atopic dermatitis (NCT03614221, NCT02669888). It may be neuroprotective against kainic acid-induced injury of the neostriatum [88] and is also under investigation for treating ulcerative colitis [89]. Most of these compounds have long safety records and are designed for chronic use, and should be considered for preclinical testing in AUD models. It should be noted that the 186 KEGG pathways included only 5266 unique genes, so the predictions should further improve as functions of genes become better understood and integrated into biological pathways.

L1000 identified more drugs targeting AUD pathways than CMap. It could be that the additional drug and cell line coverage in L1000 provides a more comprehensive catalog of drug responses. Another interpretation is that L1000 findings are spurious (false positives), which deserves consideration because most of the gene levels are imputed from the 978 measured genes in L1000. One reasonable approach to increase the chance of finding a successful AUD treatment is to select the most robust drugs to test (those that are predicted across multiple queries and databases, cell lines, doses, and time points) as suggested and implemented by us and others [22,63,90,91]. Intuitively, it seems that choosing a drug that generalizes across different conditions would bias selection towards drugs with a robust pharmacology against multiple targets. Indeed, the 8 drugs that were predicted by L1000 and CMap to oppose AUD pathways are drugs that target most pathways. One concern is that less selective drugs would have undesired side effects or toxicity, although, to the best of our knowledge, this hypothesis has not been formally tested. Studies comparing transcriptional specificity of a drug (i.e., the number of pathways or genes perturbed) to its phenotypic effects (i.e., the number of phenotypes affected by a drug and the magnitude of the effects) or side effect severity could be informative. An important finding from our analysis is that DMSO, a vehicle commonly used in behavioral pharmacology experiments, was the most pharmacologically active compound in L1000. Our results imply that experiments using DMSO as a vehicle could actually be measuring effects of a drug combination (DMSO plus the drug that was dissolved in DMSO) rather than the drug of interest alone. This could be an underappreciated reason for the lack of reproducibility across laboratories for some compounds.

There are existing databases that catalog biological pathways up- and down-regulated by compounds in the CMap database: Drug-Path (http://www.cuilab.cn/drugpath) [92] and Gene2Drug (http://gene2drug.tigem.it/) [93]. These studies only mined data available in CMap, whereas we also mined the L1000 database, greatly expanding the number of drugs and cell lines for which pathway information is now available. Another notable distinction is that Drug-Path and Gene2Drug collapse all experiments for a given drug (across various doses, cell lines, and time points) into a single list of up- and down-regulated pathways per drug, whereas we chose to analyze each signature separately. While it might be simpler to have a single list of pathways per drug, the tradeoff is a loss of information. If a researcher is predominantly interested in a single cell type, this information is available on our website and could be important as drug effects are highly cell line specific. Additionally, if multiple doses of a drug are assayed, the information we provide could hint at an effective dose, as a pathway may only be affected at higher doses of the drug. Moreover, if a pathway is affected by a compound across multiple doses and cell lines, this information could provide confidence in the prediction. We provide the frequency with which a drug affects a given pathway to make it readily apparent how often a drug targets a pathway of interest or how often a pathway is predicted to be targeted by a drug of interest.

## 5. Conclusions

In conclusion, we highlighted cell types and biological pathways that are consistently different between alcohol-dependent and control brain tissue. We mined publicly available databases to identify drugs that target these pathways in an opposing manner and highlighted eight compounds that warrant further testing. If preclinical testing of the prioritized compounds yields positive results, these compounds are already approved for other indications or are in clinical trials, and therefore, could be relatively rapidly tested in patients with AUD. 

## Figures and Tables

**Table 1:** Alcohol brain gene expression datasets.

Study	Brain Area	GEO Accession	Platform	Case/Controls
Mamdani 2015 [3]	Nucleus accumbens	GSE62699	Microarray	18 cases, 18 controls (1 female per group)
Ponomarev 2012 [4]	Central nucleus of the amygdala Basolateral amygdala Prefrontal cortex	NA	Microarray	17 cases, 15 controls (1 female per group)
Farris 2015 [5]	Prefrontal cortex	NA	RNAseq	16 cases, 15 controls (all males)
McClintick 2013 [13]	Hippocampus	GSE44456	Microarray	20 cases, 19 controls (6 females per group)
Flatscher-Bader 2008 [10]	Ventral tegmental area	GSE9058	Microarray	6 cases, 6 controls (all males)
Flatscher-Bader 2010 [9]	Nucleus accumbens	GSE20568	Microarray	10 cases, 10 controls (1 female per group)
Zhou 2011 & Farris 2015 [6,14]	Hippocampus	NA	RNAseq	8 cases, 8 controls (all males)
Zhang 2014 [11]	Prefrontal cortex	GSE49376	Microarray	23 cases, 23 controls (7 females per group)
Kapoor 2019 [7]	Prefrontal cortex	NA	RNAseq	65 cases, 73 controls (all males)
Rao 2019 [12]	Nucleus accumbens Prefrontal cortex Basolateral amygdala Central nucleus of the amygdala	NA	RNAseq	30 cases, 30 controls (7 females per group)
Mayfield	Basolateral amygdala Central nucleus of the amygdala	NA	RNAseq	17 cases (2 female) and 16 controls (1 female)

**Table 2:** Biological Pathways Disrupted in Brain Tissue from Alcohol-Dependent Patients. Pathways most commonly up-regulated (left) or down-regulated (right) in brain tissue from alcohol-dependent patients versus control. The full results are in Table S1.

Up-Regulated Pathways		Down-Regulated Pathways	
KEGG Pathway	Number of Datasets (out of 17)	KEGG Pathway	Number of Datasets (out of 17)
Complement_and_coagulation_cascades	8	Oxidative_phosphorylation	6
Focal_adhesion	8	Parkinsons_disease	4
Mapk_signaling_pathway	8	Proteasome	4
Systemic_lupus_erythematosus	8	Alzheimers_disease	3
Cytokine_cytokine_receptor_interaction	7	Cardiac_muscle_contraction	2
Ecm_receptor_interaction	7	Dna_replication	2
Cell_adhesion_molecules_cams	6	Fructose_and_mannose_metabolism	2
Leishmania_infection	6	Glycolysis_gluconeogenesis	2
Regulation_of_actin_cytoskeleton	6	Huntingtons_disease	2
Ribosome	6	Mismatch_repair	2
Leukocyte_transendothelial_migration	5	Propanoate_metabolism	2
Natural_killer_cell_mediated_cytotoxicity	5	Vibrio_cholerae_infection	2
Pathways_in_cancer	5		

**Table 3:** Biological Pathways Disrupted in Brain Tissue from Alcohol-Dependent Patients. Pathways most commonly up-regulated (top) and down-regulated (bottom) in alcohol-dependent versus control brain tissue. The brain region of the gene expression datasets is shown in the first column. The number in parentheses indicates the number of datasets that profiled gene expression in that brain region. The numbers in the table indicate the number of datasets from that brain region in which the pathway was disrupted. For example, focal adhesion was upregulated in three out of three basolateral amygdala datasets and two out of five prefrontal cortex datasets. BLA: basolateral amygdala, CNA: central nucleus of the amygdala, PFC: prefrontal cortex, NAC: nucleus accumbens, VTA: ventral tegmental area, HPC: hippocampus.

**Brain Region**	**Focal Adhesion**	**Complement and Coagulation Cascades**	**Mapk Signaling Pathway**	**Systemic Lupus Erythematosus**
BLA (3)	3	2	1	2
CNA (3)	1	2	2	1
PFC (5)	2	3	3	3
NAC (3)	1		1	
VTA (1)			1	
HPC (2)	1	1		2
**Brain Region**	**Oxidative Phosphorylation**	**Parkinsons Disease**	**Proteasome**	**Alzheimers Disease**
BLA (3)			1	
CNA (3)	1	1		1
PFC (5)	2	2	1	
NAC (3)	2	1	1	1
VTA (1)				
HPC (2)	1		1	1

**Table 4:** Prioritized List of Candidate Treatments. We identified L1000 compounds predicted to affect the top pathways perturbed in Alcohol Use Disorder in an opposing manner. We filtered the remaining compounds for those that are currently marketed or in clinical trials and most predicted to get into brain. This resulted in 37 candidate compounds. We searched the literature for each of the 37 compounds to gather information on toxicity, contraindications, and effects on neuropsychiatric traits. This resulted in a prioritized list of eight compounds in this table. MOA: mechanism of action. Launched: currently marketed compound for the given indication.

Drug	MOA	Indication	Phase
methylene-blue	guanylyl cyclase inhibitor, nitric oxide production inhibitor	methemoglobinemia	Launched
ketotifen	histamine receptor agonist, leukotriene receptor antagonist, phosphodiesterase inhibitor	itching	Launched
indirubin	CDK inhibitor, glycogen synthase kinase inhibitor		Phase 2/Phase 3
diflunisal	prostanoid receptor antagonist	rheumatoid arthritis, osteoarthritis	Launched
azelastine	histamine receptor antagonist	conjunctivitis	Launched
ALX-5407	Glycine transporter 1 inhibitor		Phase 1
rucaparib	PARP inhibitor		Phase 3
rilmenidine	adrenergic receptor agonist, imidazoline receptor agonist	hypertension	Launched

## Supplementary Materials

The following are available online at https://www.mdpi.com/2076-3425/9/12/381/s1, Figure S1: Heatmap of Cell Type Enrichment Analysis Results. Table S1: Results of the Gene Set Enrichment Analysis of KEGG pathways in the AUD brain gene expression datasets, Table S2: Results of the Cell Type Enrichment Analysis, Table S3: Results of the Gene Set Enrichment Analysis of KEGG pathways in the CMap and L1000 pharmaceutical datasets, Table S4: L1000 pharmaceuticals predicted to target the top five pathways disrupted in AUD in an opposing manner, Table S5: CMap pharmaceuticals predicted to target one or two of the top pathways disrupted in AUD in an opposing manner.
