# Supplementary material for: A Pathway-Based Genomic Approach to Identify Medications: Application to Alcohol Use Disorder
Source: Brain Sci. 2019 Dec 16;9(12):381. doi: 10.3390/brainsci9120381 (PMC6956180; doi:10.3390/brainsci9120381)
Supplement: Supplementary file 1 [file brainsci-09-00381-s001.zip › FigS1_heatmaps_cell_type_enrichment_with_legend.pdf]

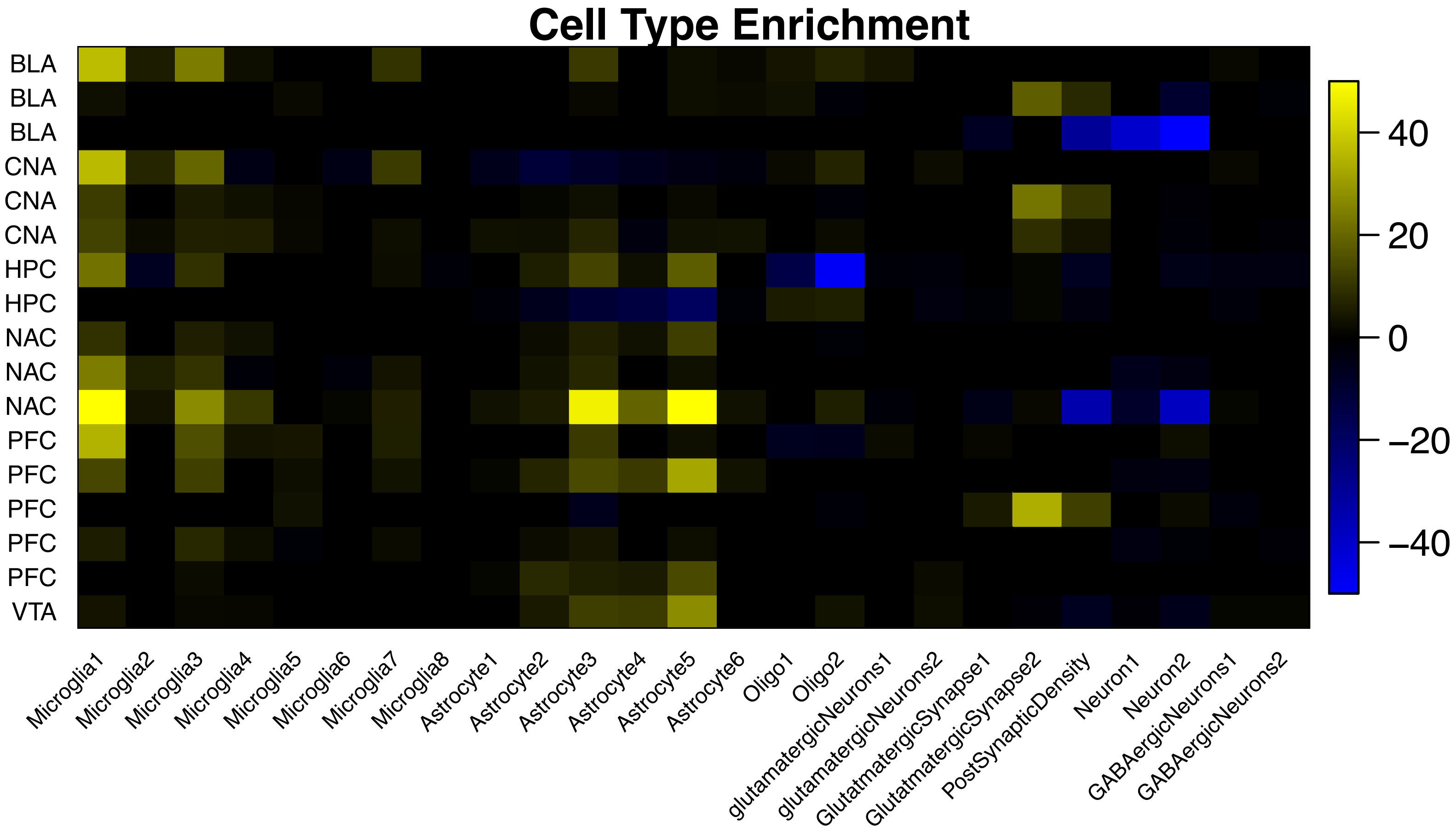

Figure S1. Heatmap displaying the $\log_{10}$p-value associated with the enrichment of cell type specific datasets within the genesets up-regulated (yellow) or down-regulated (blue) in human alcoholic brain tissue. The brain area for the human gene expression study is shown (BLA: basolateral amydala, CNA: central nucleus of the amygdala, HPC: hippocampus, NAC: nucleus accumbens, PFC: prefrontal cortex, VTA: ventral tegmental area). Table S2 includes all p values and the full names of the cell type datasets. See Methods for details. More information on cell type datasets can be found here: https://www.rdocumentation.org/packages/WGCNA/versions/1.25-1/topics/userListEnrichment/.
